# Therapies go digital. What drives physicians’ acceptance?

**DOI:** 10.1371/journal.pone.0303302

**Published:** 2024-05-10

**Authors:** Alessandro Carrera, Emanuele Lettieri, Gaia Lietti, Sara Martignoni, Chiara Sgarbossa, Joseph Cafazzo

**Affiliations:** 1 Department of Management Engineering, Politecnico di Milano, Milano, Italy; 2 Institute of Health Policy, Management and Evaluation, University of Toronto, Toronto, Ontario, Canada; Northwestern University, UNITED STATES

## Abstract

National healthcare systems face multiple challenges, including the increasing demand for care and decreasing availability of healthcare professionals. Digital health technologies represent opportunities that offer improved efficiency, accessibility, and patient care. In this scenario, Digital Therapeutics are technological advancements to treat or alleviate a disease and deliver a medical intervention with evidence-based therapeutic impacts and regulatory approval. Digital Therapeutics are a paradigm shift for physicians, who exercise caution in terms of trust and wide usage. Digital Therapeutics represents an opportunity and a challenge in healthcare system integration. The research investigates the factors explaining physicians’ acceptance of Digital Therapeutics. A research model that combines organizational mechanisms derived from Institutional Theory and rational factors derived from the Technology Acceptance model was developed. The model was tested through 107 responses from a survey distributed to the members of the leading Italian scientific society in Diabetology. Literature-based hypotheses were empirically tested through Structural Equation Modelling. The analysis confirmed the influence of Perceived Ease of Use on Perceived Usefulness and Perceived Usefulness on the Intention To Use Digital Therapeutics. Rules and norms impact Perceived Usefulness when considering the influence of the scientific society. Culture and mindset towards innovation within the hospital positively affect Perceived Ease of Use. The readiness of hospital facilities enhances the extent to which physicians perceive the ease of employing Digital Therapeutics in their daily practice. Instead, esteemed colleagues’ opinions and guidelines from the scientific society reveal to physicians the value of Digital Therapeutics in patients’ care pathways. Institutions should prioritize cultural, normative, and regulative aspects to accelerate physicians’ endorsement of Digital Therapeutics. Findings advance the theoretical knowledge around clinicians’ adoption of innovative digital health technologies, unveiling the interaction between rational and institutional factors. The results highlight practical implications for healthcare institutions and Digital Therapeutics manufacturers willing to promote their adoption.

## 1. Introduction

In the rapidly changing landscape of the life sciences sector, digital transformation has emerged as a crucial force reshaping how healthcare is delivered and accessed [1–3]. Digitalization of healthcare systems is becoming increasingly compelling due to the critical confluence of increasing demand for care and decreasing availability of healthcare professionals [4,5]. National healthcare systems worldwide are challenged to address these vital issues as they adapt to this evolving paradigm [6]. Key challenges include integrating innovative solutions to meet the growing healthcare needs of patients and citizens [7,8]. In this changing scenario, digital health technologies represent both an opportunity and a challenge [9]. Indeed, they offer, on the one hand, the perspective of efficiency, accessibility, and improved patient care [10,11]. At the same time, these innovations must be understood and integrated into existing care pathways and clinical practice [12].

One notable example of digital health technology deserving profound attention is represented by Digital Therapeutics (DTx). International Organization for Standardization (ISO) recently defined DTx as a category of evidence-based software programs designed to prevent and manage medical conditions [13]. DTx have emerged as a powerful tool among the latest digital health technologies [14]. These digital interventions hold the potential to alleviate the burden on healthcare systems by effectively supplementing clinical care [15]. DTx are often considered software medical devices that must be prescribed by physicians [16]. Therefore, for DTx to demonstrate their potential, they must find a place in the practices of healthcare providers, particularly physicians [17]. Their acceptance and integration into the healthcare framework represent a pivotal milestone in adopting and diffusing these technologies [18]. Indeed, it has been observed that the role of physicians is crucial in the distribution process of DTx [19]. Therefore, the spread of DTx is strongly linked to the knowledge and subsequent acceptance by healthcare professionals [20]. These recent experiences underscore the importance of considering physicians’ views and understanding the elements that can lead to the effective use and adoption of DTx [21]. Factors such as workload pressure [22], lack of comprehensive regulatory framework [22], limited evidence on outcomes [22,23], low digital proficiency [24], and technological failures [25,26] might hinder the adoption of novel solutions. Therefore, being well-informed of the benefits and mechanisms of a digital solution is vital to fostering the adoption rate among end users, especially for clinicians [25,27].

The literature on clinicians’ intention to use new technologies appears well-established. The Technology Acceptance Model (TAM) remains an extraordinarily relevant and applied theory for evaluating the choice to use technology by considering the most rational factors underlying the adoption process [28,29]. However, although the TAM and its extensions have been applied to analyze physicians’ acceptance of digital health technologies (e.g., telemedicine [30] and electronic medical records [31]), further elements should be considered when dealing with disruptive technologies like DTx to understand the users’ intention to use them fully [32,33]. In the realm of organizational decision-making, an alternative viewpoint challenges the conventional idea that decisions are solely driven by rational evaluations aimed at optimizing efficiency and effectiveness, highlighting the necessity of considering irrational elements arising from the complex dynamics within the organizational environment [34]. In this scenario, Institutional Theory has been successfully applied to several studies on the propensity to use new technologies. This approach enables researchers to capture the more irrational components underlying individual choices not fully included in the TAM [35,36].

This study aims to understand better the interplay between institutional factors and rational elements and their degree of influence on the decision-making process about physicians’ technology adoption. In other words, this research intends to build a framework to analyze the main determinants influencing physicians’ acceptance of Digital Therapeutics relying on the combination of Institutional Theory and TAM.

The article is organized as follows. Section 2 summarizes the design of the proposed model. Then, the methodology, data collection, and analysis are presented. Section 3 reports the results of the empirical investigation. Section 4 elaborates on the findings, highlighting the main theoretical and practical implications and suggesting possible future developments. Finally, Section 5 summarizes the outcome of the research.

## 2. Materials and methods

### 2.1. Model design

The evaluation of relevant insights from studies on the intentional behavior of medical professionals towards innovation identified two significant factors that influence their decision-making. The first factor is the rational evaluation, which focuses on optimizing efficiency and effectiveness based on individual perception. Second, scholars highlight institutional factors impacting physicians’ behavior, such as the organization’s policies and structure. Personal characteristics, competencies, psychological attributes, and values significantly determine physicians’ willingness to adopt new technologies. Previous studies have explored various domains related to work, technology awareness, digital literacy, and the generation gap, providing valuable insights into physicians’ behavior toward innovation.

**Fig 1:**
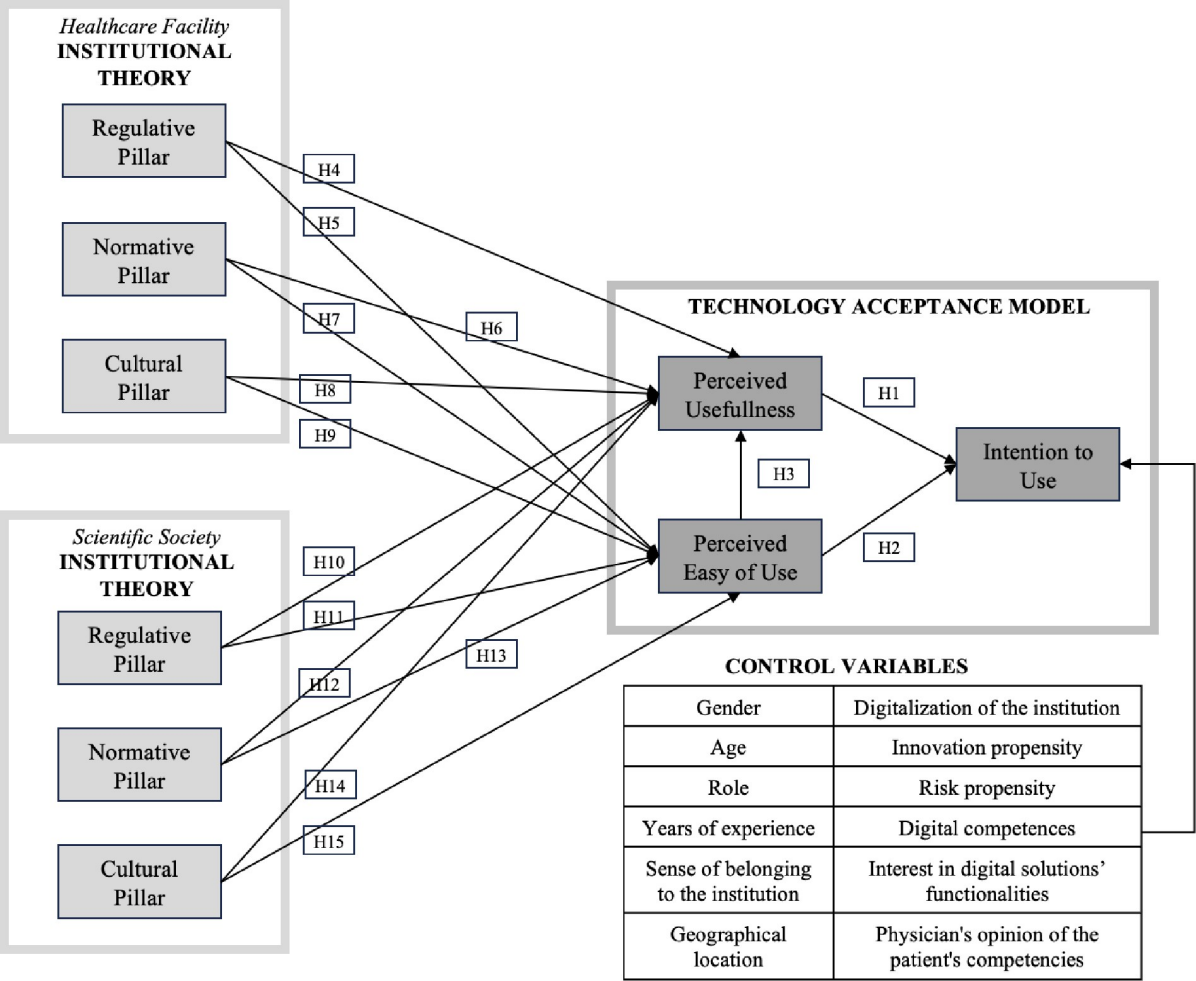
Proposed model and hypotheses (H).

The Technology Acceptance Model summarizes rational evaluation, while the Institutional Theory investigates institutional factors. TAM aims to assess the Intention to Use (ITU) by determining the Perceived Usefulness (PU) and Perceived Ease of Use (PEU) to explain rational elements influencing physicians’ intentional behavior to embrace innovative solutions [28]. On the other hand, institutions are comprised of Regulative (RP), Normative (NP), and Cultural (CP) pillars profoundly affecting rational evaluations and driving actors’ decisions [35]. The literature points out that the aspects considered by both theories are crucial in adopting a new technology. In the medical field, organizational factors, dynamics within healthcare teams, and the benefits of information sharing were found to be important in adoption decisions. Therefore, the institutional context in the case of physicians is referred to both the healthcare facility and the relevant scientific society. Indeed, the working environment of physicians is strongly characterized by the presence of these two institutions, whose rules and dynamics assume a central role in the clinical practice of professionals. The combination of the two theories has not been studied extensively in the literature. This study develops a new model that considers the relationship between TAM and Institutional Theory and measures their impact on physicians’ intention to use a DTx (Fig 1).

The research model includes the fundamental hypotheses (H) of the TAM [29,37] and six additional assumptions for each institution considered [36,38]. PU is forecasted to positively impact ITU (H1)^24,32,34^, together with PEU (H2) [28,37,39,40], and PEU is expected to affect PU (H3) positively [37,41]. Moreover, the organizational elements are envisioned to be antecedents of both PU and PEU. Specifically, RP is hypothesized to be positively linked to PU (H4, H10) [42,43], as the construct can impact physicians’ perception of benefits and their possibility to exploit technological advancements in compliance with the regulative framework. RP is also related to PEU (H5, H11) [42,43] since clinicians are likely to regard the established rules as instructive guidelines for usage. NP affects PU (H6, H12) [36,44,45] through the influence of peers who work in the same hospital facility or refer to the same scientific society. Observing collogues using and gaining advantages from digital solutions leads professionals to anticipate comparable outcomes. This analogous influence also impacts PEU (H7, H13) [36,44,45] by drawing on the experiences of other clinicians. Finally, CP is positively connected to PU (H8, H14), assuming the influence on the doctors’ propensity to perceive the technology as applicable and appropriate [46,47]. Finally, the positive culture (CP) within institutions is also envisioned to affect PEU (H9, H15) by fostering the inclination of medical professionals toward technological progress [46,47].

Also, relying on past literature, 12 control variables were included to enhance the validity and reliability of research outcomes [48]. Control variables include gender [39,49,50], age [24,49,51], years of experience, role, sense of belonging to the institution, geographical location [52], level of digitalization of the institution [53], innovation propensity [54], risk propensity [55,56], digital competencies [22,51], interest towards digital technology [57,58], and opinion of the patients’ competencies [59,60].

### 2.2. Data collection

The validation of the model relies on a self-administered web-based survey available through an online version. The web-based questionnaire is preferred for its accessibility, cost-efficiency, and convenience in data collection [61]. Moreover, a self-administered survey allows respondents to answer at their convenience, disregarding location and time [62]. The survey was designed in compliance with the General Data Protection Regulation (GDPR) to maintain respondents’ anonymity, thereby ensuring their total freedom to express opinions and beliefs while minimizing the potential for biased responses [63]. Participants gave written informed consent to participate in this study, agreeing to the purposes and methods of data processing at the outset, making this explicit at the beginning of the questionnaire.

Being chronic diseases the primary target for the technology under examination, healthcare practitioners within this specialty are a pragmatic and realistic cohort for the study. Specifically, a growing interest in DTx suppliers is recorded in the endocrinological field for the treatment of diabetes [64,65]. Already approved DTx in this field are currently available on the market (e.g. BluestarRx) [66,67]. Therefore, the research was conducted in collaboration with Italy’s leading diabetes scientific association whose perspective serves as crucial insights for understanding clinicians’ acceptance of such technologies. Italy is characterized by a public health service and the absence of specific regulations on DTx, making the country an interesting context for analysis. Besides playing a pivotal role in facilitating the questionnaire distribution, the organization provided valuable insights during the survey development and approval phases. Different contact persons are considered to obtain valuable feedback.

One hundred fifty-eight responses were registered from the survey distributed to the clinicians and subsequently analyzed. Since the questionnaire was delivered to diabetologists, the DTx example provided was consistent with their specialty to increase the understandability of questions. The survey is made of 61 questions divided into three parts measuring demographic information (Part A), theoretical constructs (Part B), and control variables (Part C) of the proposed model. All the items have been measured through a 5-point Likert scale, thereby contributing to the overall integrity and accuracy of the findings [68]. Additional information is available in S1 and S2 Appendices.

### 2.3. Data analysis

**Fig 2:**
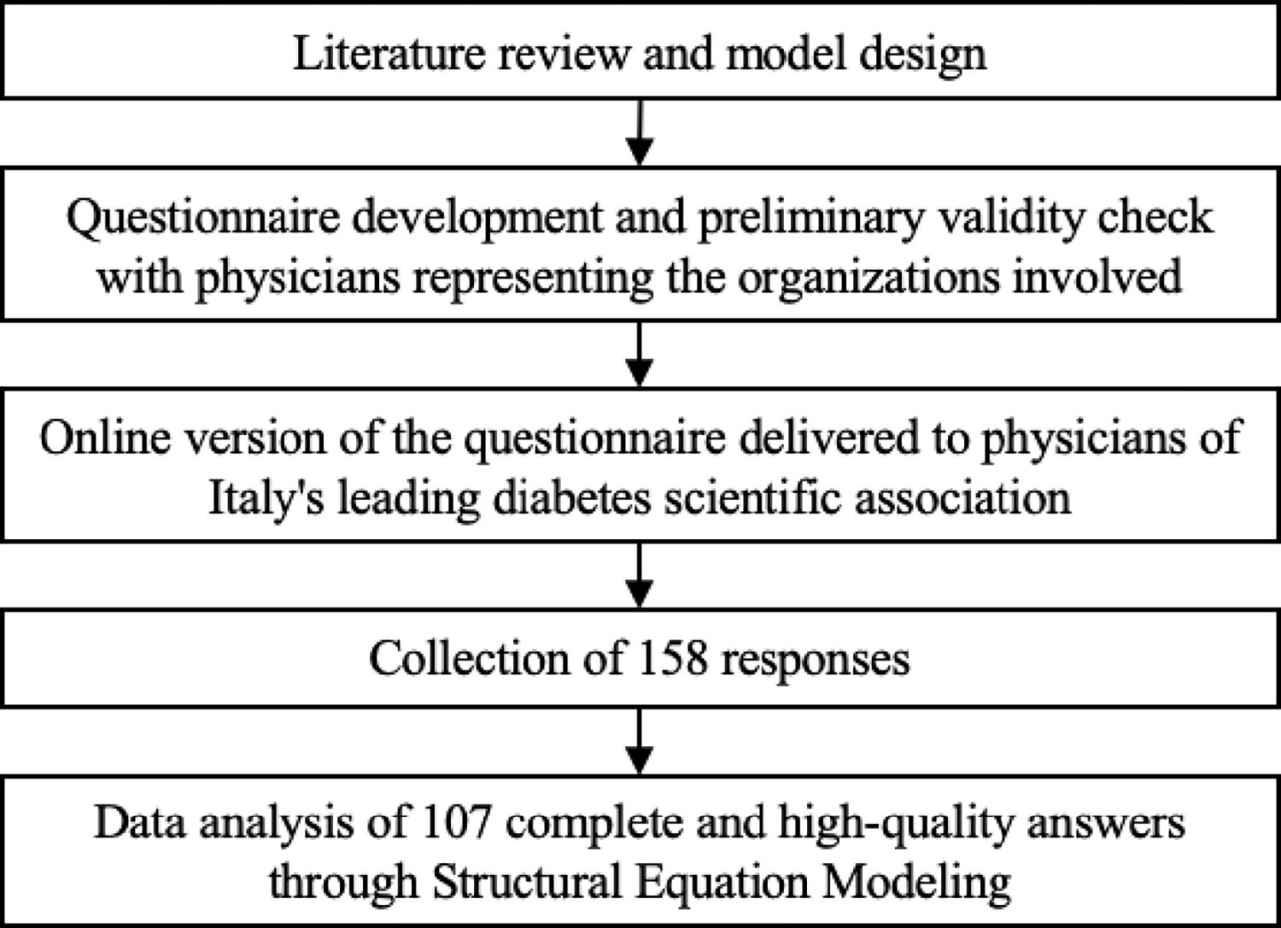
Overview of the methodology followed.

A total of fifty-one responses were disregarded because they were either blank or incomplete in the questionnaire sections. Therefore, the analysis relied on 107 high-quality (e.g., complete) responses. The collected data were first analyzed by descriptive analysis of demographic and personal information questions. Second, the model was tested using STATA 17 software through Structural Equation Modeling (SEM), an effective analytical tool in health systems studies [69]. A summary of the methodology followed for this research is shown in Fig 2.

The dataset was tested to verify the absence of biases employing Harman’s Single Factor Test [70]. Then, the Kaiser-Meyer-Olkin (KMO) test was applied to verify the sample adequacy for the factor analysis [71]. Moreover, literature-based hypotheses were tested, and Cronbach’s alphas ensured validity and consistency [72]. Lastly, the goodness of fit (GOF) was verified [73]. Analyses included both absolute, like the square error of approximation (RMSEA) and the standardized root mean residual (SRMR), and incremental indicators, like the comparative fit index (CFI) and the Tucker-Lewis index (TLI).

## 3. Results

This section summarizes the results obtained based on responses to the questionnaire.

Before proceeding with model validation, several quantitative analyses were conducted to confirm the dataset’s and sample’s adequacy. The Harman’s Single Factor Test returned a value of 0.3925 (< 0.5), ensuring no bias related to the instrument used for data collection [70]. The KMO test returned a value of 0.8541, greater than 0.8, indicating adequate sampling [71]. Cronbach’s alphas were computed, and constructs’ reliability and internal consistency were assessed (Table 1) [74].

**Table 1:** Constructs, measurement items, and calculation of Cronbach’s alphas for the proposed model.

Construct	Item	Measurement item	Factor loading	Cronbach’s α
**Intention To Use (ITU)**	ITU1	I would like to use this DTx	0.8350	0.9198
	ITU2	I intend to regularly use this DTx in my work	0.8197	
	ITU3	I would be inclined to use this DTx	0.8265	
**Perceived Usefulness (PU)**	PU1	Using this DTx would optimize the way I work	0.7702	0.9356
	PU2	Using this DTx would allow me to better manage my patients’ treatment journey	0.7838	
	PU3	Using this DTx would improve the quality of my work	0.7712	
	PU4	Using this DTx would increase the effectiveness of my work	0.8354	
**Perceived Ease of Use (PEU)**	PEU1	I think that the use of this DTx would not require me to make a great effort	0.5404	0.8743
	PEU2	I think the interface of this DTx would be clear, understandable, and intuitive to me	0.6786	
	PEU3	I think I would have no difficulty using the different functionalities of this DTx on my smartphone	0.6241	
**Healthcare Facility Regulative Pillar (RP1)**	RP1.1	If I tried to use this DTx in the healthcare facility where I work, I would run up against the procedures in place today	0.0046	0.7316
	RP1.2	Some rules in place today in the healthcare facility where I work would prevent me from using this DTx effectively	-0.1975	
	RP1.3	The regulations I follow today within the healthcare facility where I work would not allow me to use this DTx	-0.1338	
**Healthcare Facility Normative Pillar (NP1)**	NP1.1	I think the colleagues I value most within the healthcare facility where I work would consider the use of this DTx appropriate	0.6390	0.8019
	NP1.2	The colleagues I value most within the healthcare facility where I work would think it would be interesting and beneficial to use this DTx	0.6268	
	NP1.3	The colleagues I value most within the healthcare facility where I work would NOT think I would waste my time using this DTx	0.5501	
**Healthcare Facility Cultural Pillar (CP1)**	CP1.1	In the healthcare facility where I work, there is full confidence in digital innovation (e.g., this DTx)	0.5024	0.9257
	CP1.2	In the healthcare facility where I work, there is full openness to trying new digital solutions (e.g., this DTx)	0.4552	
	CP1.3	The healthcare facility where I work is totally open to the introduction of digital solutions (e.g., this DTx)	0.3880	
**Scientific Society Regulative Pillar (RP2)**	RP2.1	If I used this DTx, my scientific reference society would have doubts about consistency with the procedures it recommends	-0.1861	0.8375
	RP2.2	Some rules promoted by my scientific reference society would prevent me from using this DTx	-0.4773	
	RP2.3	The regulations I follow within my scientific reference society would NOT allow me to use this DTx	-0.4682	
**Scientific Society Normative Pillar (NP2)**	NP2.1	I think the colleagues I value most within my scientific reference society would consider the use of this DTx appropriate	0.6335	0.8430
	NP2.2	The colleagues I value most within my scientific reference society would think it would be interesting and beneficial to use this DTx	0.7411	
	NP2.3	The colleagues I value most within my scientific reference society would NOT think I would waste my time using this DTx	0.6560	
**Scientific Society Cultural Pillar (CP2)**	CP2.1	There is full confidence in digital innovation (e.g., this DTx) in my scientific reference society	0.7405	0.9571
	CP2.2	In my scientific reference society, there is full openness to try new digital solutions (e.g., this DTx)	0.7521	
	CP2.3	My scientific reference society is totally open to the introduction of digital solutions (e.g., this DTx)	0.7406	

SEM was employed to test the hypotheses. Table 2 shows the results. Concerning the rational factors, the relations between PU and ITU (p-value of 0.000) and PEU and PU (p-value of 0.000) were confirmed. Indeed, it is reasonable to deduce that the usefulness of DTx perceived by healthcare practitioners, encompassing the potential for deriving benefits from its employment, can positively influence their willingness to embrace it. Moreover, the perceived effectiveness of the digital solution is enhanced by the simplicity of its usage. Accordingly, the easier is to employ DTx, the more benefits the technology offers for patient management. Within the realm of healthcare facilities, the influence of CP1 on PEU demonstrates statistical significance (p-value of 0.008). Physicians, due to the continuous interaction with their working environment, tend to develop personal considerations increasingly consistent with the organization itself. In the case of DTx, the influence of the healthcare facility may shape physicians’ perceived simplicity in usage. Further, if DTx adoption aligns with the hospital’s mindset, clinicians find it less complex and complicated to approach these technologies. Regarding the scientific reference society’s context, statistical relevance between RP2 and PU is observed (p-value of 0.043). The findings suggest that a well-structured, monitored, and esteemed regulatory framework can foster trust and, consequently, enhance perceived benefits. Out of the analysis results, the hypothesized effect of NP2 on PU exhibits statistical significance (p-value of 0.029). Hence, it is proven that the perception of DTx effectiveness is enhanced by the opinion of esteemed colleagues belonging to the same association.

**Table 2:** Results of path analysis and hypothesis testing of model constructs.

Hypothesis	Path	β Coef.	Standard error	*p*-value
**H1**	PU → ITU	0.7495237	0.0842045	0.000***
**H2**	PEU → ITU	0. 1055515	0.0737387	0.152
**H3**	PEU → PU	0.4345539	0.1016312	0.000***
**H4**	RP1 → PU	-0.0586822	0.1053932	0.578
**H5**	RP1 → PEU	0.0133258	0.1319658	0.920
**H6**	NP1 → PU	0.2041237	0.1180295	0.084
**H7**	NP1 → PEU	0.1945438	0.1466334	0.185
**H8**	CP1 → PU	-0.0826558	0.0949037	0.384
**H9**	CP1 → PEU	0.3116152	0.117704	0.008**
**H10**	RP2 → PU	-0.2955777	0.1463163	0.043*
**H11**	RP2 → PEU	0.157668	0.1752278	0.368
**H12**	NP2 → PU	0.4416643	0.2025694	0.029*
**H13**	NP2 → PEU	0.2049883	0.2535029	0.419
**H14**	CP2 → PU	0.0148225	0.1463525	0.919
**H15**	CP2 → PEU	0.3177102	0.1820423	0.081

Among the control variables, the Age (*p*-value of 0.037), the Role (*p*-value of 0.050), the Sense of belonging to the scientific society (*p*-value of 0.039), and Risk Propensity (*p*-value of 0.027) of physicians emerged as statistically significant in influencing the ITU (Table 3).

**Table 3:** Incidence of control variables on intention to use.

Control variable	β Coef.	Standard error	*p*-value
**Age**	-0.1362096	0.0652571	0.037*
**Role**	0.1262086	0.065644	0.050*
**Sense of belonging to the scientific reference society**	0.2735228	0.1325357	0.039*
**Risks propensity**	-0.4013708	0.1818262	0.027*

Finally, all statistics considered to measure the model’s goodness of fit met the thresholds [73], as shown in Table 4.

**Table 4:** The goodness of fit indexes of the proposed model.

Indicator	Threshold	Value
**RMSEA**	< 0.08	0.076
**SRMR**	< 0.08	0.078
**CFI**	> 0.9	0.920
**TLI**	> 0.9	0.908

## 4. Discussion

This section presents a discussion related to the interpretation of the results of this study. The research provides an original contribution to the body of academic literature and, at the same time, produces several practical and managerial insights.

### 4.1. Theoretical contribution

Research findings significantly advance the theoretical understanding of professionals adopting innovative digital health technologies by delving into the little-explored relationship between rational and institutional factors. From this perspective, the proposed model helps when it comes to physicians. Indeed, the healthcare facility they work in and the scientific society they refer to are relevant to the new innovative technology process. It was indeed possible to observe how all three pillars of the Institutional Theory positively influence the rational factors that lead a physician to use a DTx. Indeed, the readiness of the healthcare facility and openness to the introduction of innovation within it (CP1) increases the perception that a DTx is undoubtedly easy to use. The absence of additional resistance in the culture of the work environment impacts the intention to use new technology in clinical practice.

On the other hand, protocols (RP2) and colleagues (NP2) views within the same scientific society influence the individual physicians’ perception of the usefulness of an innovation. Clinicians in the same scientific community, in fact, share experiences and training on an ongoing basis concerning how to innovate and improve the care of their patients. Association guidelines, experience, and advice from respected colleagues influence the perceived level of usefulness related to the use of a DTx. When physicians believe a DTx is useful (PU), they are likelier to use it (ITU).

### 4.2. Managerial contribution

The study also provides insights with practical implications. Hospital readiness for new technologies and digital innovation is crucial in influencing how physicians perceive the ease of integrating DTx into their daily practice. When healthcare facilities are well prepared and equipped to support these technologies, physicians are more likely to view them as easy-to-use and practical tools for patient care. In addition, DTx adoption by clinicians is influenced by the opinions of respected colleagues, especially if they have direct experience with digital health technologies. Moreover, the guidance provided by scientific societies can impact clinicians’ intention to use DTx.

Physicians often value the insights and recommendations of their colleagues and professional organizations when considering the adoption of new technologies, such as Digital Therapeutics for patient care. In addition, recognizing that the role physicians hold within the hospital setting exerts influence on the technology adoption process is crucial. Distinct job responsibilities necessitate different tools, and the choice to integrate DTx is directly related to how the digital solution aligns with the specific requirements of the institutional position covered by clinicians. Moreover, physicians’ intentional behavior is directly affected by the sense of affiliation to their scientific community. Thus, medical associations should maximize the level of involvement in their initiatives to guarantee a successful dissemination of technological knowledge.

To promote physician adherence to DTx, institutions such as hospitals and scientific societies should prioritize cultural, normative, and regulatory aspects. These factors create an environment favorable to the physicians’ adoption of these technologies by aligning them with established norms, values, and regulations within the healthcare system. This strategic approach can accelerate the adoption process of DTx and lead to better patient care.

Lastly, results uncover primary features for DTx development, enabling the solution to bring a disruptive impact on the healthcare field. Providing clinical evidence mitigates the risk embedded within the introduction of the solution in medical treatments. Additionally, a more secure and compliant environment fosters physicians’ confidence and enables the diffusion process. The seniority of adopters is a further noteworthy aspect to consider within the DTx design phase. When deploying new medical devices, different needs and capabilities across physicians’ age clusters should be considered to guarantee a successful endorsement.

### 4.3. Limitations and future research

The research is not exempt from limitations, which can be considered a starting point for further study. First, although representative of a highly relevant category of physicians in Digital Therapeutics, the sample is currently limited. This inevitably impacts the possibility of generalizing the results. In this regard, the study could be expanded by future research aiming to include additional medical specialties and another country to conduct further analysis to obtain other insights and comparisons, drawing more robust conclusions. In addition, future research could better explore the role of institutions by emphasizing how they influence physicians. This study aimed to determine whether such influences existed. Therefore, it would be interesting to address this issue, taking advantage of a qualitative approach that could complement quantitative research.

Finally, this study focuses on factors that may explain physicians’ acceptance of DTx. The relevance of the physician’s role stems from the fact that, at present, the most widespread model of DTx distribution internationally (e.g., in Germany, and France) involves a prescription by the physician [19,75]. However, these technologies should work to minimize the efforts of highly specialized, rationed specialists [76]. It is therefore worth asking whether it might be useful to develop different implementation models in the future. Future research could systematically address these issues, assessing the advantages and limitations of different models to foster the diffusion of these innovative solutions, starting with the factors that drive clinicians to use Digital Therapeutics.

## 5. Conclusions

This study sheds new light on the unexplored interplay between rational and institutional elements in physician acceptance of a new digital health technology. The findings significantly improve the theoretical understanding of physicians adopting innovations such as Digital Therapeutics to treat chronic diseases. In addition, this study underscores the practical importance of these findings, offering valuable insights for healthcare institutions and manufacturers of Digital Therapeutics seeking to facilitate their adoption. Completing the research with additional data will enable greater generalization of the findings.

## Supporting information

S1 Appendix Questionnaire submitted to clinicians. (DOCX)

S2 Appendix Dataset collected from the survey. (PDF)
