# Supplementary material for: Therapies go digital. What drives physicians’ acceptance?
Source: PLoS One. 2024 May 10;19(5):e0303302. doi: 10.1371/journal.pone.0303302 (PMC11086840; doi:10.1371/journal.pone.0303302)
Supplement: S1 Appendix — (DOCX) [file pone.0303302.s001.docx]

## S1 Appendix

This section contains the questionnaire submitted to clinicians. The questionnaire was adapted to the medical specialty by providing examples of Digital Therapeutics that are coherent with clinicians' fields (namely diabetes). In more detail, the questionnaire is made in three sections: Part A (demographics and institutions information), presented in Table A; Part B (theoretical construct measurement), presented in Table B; and Part C (control variables measurement), shown in Table C. Part B and Part C have been measured through a Likert Scale from 1: strongly disagree to 5: strongly agree.

**Table A. Part A of the submitted questionnaire.** Demographics and institutions information.

| # | Question | Answer |
| --- | --- | --- |
| 1 | Gender | Male |
|  |  | Female |
|  |  | I prefer not to answer |
| 2 | Age | < 30 |
|  |  | 30-39 |
|  |  | 40-49 |
|  |  | 50-59 |
|  |  | 60-69 |
|  |  | >70 |
| 3 | Role | Department director |
|  |  | Facility director (simple/complex) |
|  |  | Medical doctor |
|  |  | Internal outpatient specialist |
|  |  | Freelancer |
| 4 | Years of experience in your current role | < 5 years |
|  |  | 10 years |
|  |  | 20 years |
|  |  | 30 years |
|  |  | > 30 years |
| 5 | How frequently do you take part in initiatives and/or events organized by your scientific reference society? | Never |
|  |  | Rarely |
|  |  | Often |
|  |  | Always |
| 6 | The region where you work | Select one among all the Italian regions |
| 7 | Does the facility where you work have an Electronic Medical Record (EHR) in place? | Yes, it does, and I personally use it |
|  |  | Yes, it does but I personally don't use it |
|  |  | No, it doesn't |
|  |  | I don't know |

**Table B. Part B of the submitted questionnaire.** Measurement of the theoretical constructs of the proposed model through Likert Scale (from 1: strongly disagree to 5: strongly agree).

| # | Question | 1 | 2 | 3 | 4 | 5 |
| --- | --- | --- | --- | --- | --- | --- |
| 8 | I would like to use this DTx |  |  |  |  |  |
| 9 | I intend to regularly use this DTx in my work |  |  |  |  |  |
| 10 | I would be inclined to use this DTx |  |  |  |  |  |
| 11 | Using this DTx would optimize the way I work |  |  |  |  |  |
| 12 | Using this DTx would allow me to better manage my patients' treatment journey |  |  |  |  |  |
| 13 | Using this DTx would improve the quality of my work |  |  |  |  |  |
| 14 | Using this DTx would increase the effectiveness of my work |  |  |  |  |  |
| 15 | I think that the use of this DTx would not require me to make a great effort |  |  |  |  |  |
| 16 | I think the interface of this DTx would be clear, understandable, and intuitive to me |  |  |  |  |  |
| 17 | I think I would have no difficulty using the different functionalities of this DTx on my smartphone |  |  |  |  |  |
| 18 | If I tried to use this DTx in the healthcare facility where I work, I would run up against the procedures in place today |  |  |  |  |  |
| 19 | Some rules in place today in the healthcare facility where I work would prevent me from using this DTx effectively |  |  |  |  |  |
| 20 | The regulations I follow today within the healthcare facility where I work would not allow me to use this DTx |  |  |  |  |  |
| 21 | I think the colleagues I value most within the healthcare facility where I work would consider the use of this DTx appropriate |  |  |  |  |  |
| 22 | The colleagues I value most within the healthcare facility where I work would think it would be interesting and beneficial to use this DTx |  |  |  |  |  |
| 23 | The colleagues I value most within the healthcare facility where I work would NOT think I would waste my time using this DTx |  |  |  |  |  |
| 24 | In the healthcare facility where I work, there is full confidence in digital innovation (e.g., this DTx) |  |  |  |  |  |
| 25 | In the healthcare facility where I work, there is full openness to trying new digital solutions (e.g., this DTx) |  |  |  |  |  |
| 26 | The healthcare facility where I work is totally open to the introduction of digital solutions (e.g., this DTx) |  |  |  |  |  |
| 27 | If I used this DTx, my scientific reference society would have doubts about consistency with the procedures it recommends |  |  |  |  |  |
| 28 | Some rules promoted by my scientific reference society would prevent me from using this DTx |  |  |  |  |  |
| 29 | The regulations I follow within my scientific reference society would NOT allow me to use this DTx |  |  |  |  |  |
| 30 | I think the colleagues I value most within my scientific reference society would consider the use of this DTx appropriate |  |  |  |  |  |
| 31 | The colleagues I value most within my scientific reference society would think it would be interesting and beneficial to use this DTx |  |  |  |  |  |
| 32 | The colleagues I value most within my scientific reference society would NOT think I would waste my time using this DTx c |  |  |  |  |  |
| 33 | There is full confidence in digital innovation (e.g., this DTx) in my scientific reference society |  |  |  |  |  |
| 34 | In my scientific reference society, there is full openness to try new digital solutions (e.g., this DTx) |  |  |  |  |  |
| 35 | My scientific reference society is totally open to the introduction of digital solutions (e.g., this DTx) |  |  |  |  |  |

**Table C. Part C of the submitted questionnaire.** Measurement of the control variables of the proposed model through Likert Scale (from 1: strongly disagree to 5: strongly agree).

| # | Question | 1 | 2 | 3 | 4 | 5 |
| --- | --- | --- | --- | --- | --- | --- |
| 36 | I keep myself informed about the latest developments in digital solutions in my area of interest |  |  |  |  |  |
| 37 | I am eager to learn about new digital solutions applicable to the context in which I work |  |  |  |  |  |
| 38 | Usually, I am one of the first among my acquaintances to become aware of new digital solutions |  |  |  |  |  |
| 39 | I am inclined to accept the clinical risk associated with using a new digital solution compared with an established treatment |  |  |  |  |  |
| 40 | I am willing to accept the clinical risk associated with using a new digital solution to improve the patient care pathway |  |  |  |  |  |
| 41 | I am inclined to accept the clinical risk associated with using a new digital solution to improve clinical practice |  |  |  |  |  |
| 42 | Using DTx would pose risks related to privacy and protection of collected clinical data |  |  |  |  |  |
| 43 | Using DTx would pose risks due to misdiagnosis and poor clinical decisions |  |  |  |  |  |
| 44 | Using DTx would pose risks related to the impairment of the doctor-patient relationship |  |  |  |  |  |
| 45 | Using DTx would pose risks due to the lack of a high level of clinical evidence |  |  |  |  |  |
| 46 | Using DTx would pose risks due to the incompleteness of the regulatory framework |  |  |  |  |  |
| 47 | Using DTx would pose risks due to my low level of digital skills needed to know how it works |  |  |  |  |  |
| 48 | Using DTx would pose risks due to the failure of technological aspects |  |  |  |  |  |
| 49 | I believe that I possess the skills necessary to use smartphones |  |  |  |  |  |
| 50 | I believe that I possess the skills necessary to use smartphone applications to manage personal data (e.g., home banking) |  |  |  |  |  |
| 51 | I believe that I possess the skills necessary to use digital identity systems (e.g., SPID) |  |  |  |  |  |
| 52 | I believe that I possess the skills necessary to exchange data, information, and documents via digital tools securely and in compliance with privacy requirements |  |  |  |  |  |
| 53 | I believe that I possess the skills necessary to store data, information, and documents securely and in compliance with privacy requirements |  |  |  |  |  |
| 54 | I believe that I possess the skills necessary to analyze data through graphs and understand them |  |  |  |  |  |
| 55 | I believe that I possess the skills necessary to communicate with patients and caregivers through digital tools |  |  |  |  |  |
| 56 | I believe that I possess the skills necessary to communicate with other healthcare professionals through digital tools |  |  |  |  |  |
| 57 | I believe that I possess the skills necessary to use different technologies consciously and carefully while balancing productivity and work-life balance |  |  |  |  |  |
| 58 | I am interested in using a digital solution that increases patient adherence to therapy |  |  |  |  |  |
| 59 | I am interested in using a digital solution that supports me in decision-making through patient data collection and analysis (e.g., dosage of drugs) |  |  |  |  |  |
| 60 | I would be inclined to recommend a digital solution if I believed that my patients had the necessary digital tools (e.g., smartphones, internet connection) for its use |  |  |  |  |  |
| 61 | I would be inclined to recommend a digital solution if I believed that my patients had the necessary digital skills for its use or had the opportunity to develop them |  |  |  |  |  |
