# Supplementary material for: Therapies go digital. What drives physicians’ acceptance?
Source: PLoS One. 2024 May 10;19(5):e0303302. doi: 10.1371/journal.pone.0303302 (PMC11086840; doi:10.1371/journal.pone.0303302)
Supplement: S2 Appendix — (PDF) [file pone.0303302.s002.pdf]

GEN	AGE	ROLE	YoE	SRS	REG	EHR	ITU1	ITU2
2	3	2	1	4	5	1	4	5
2	3	2	1	4	16	1	4	4
2	2	4	1	3	9	1	4	4
2	2	3	2	3	12	1	4	4
2	3	4	3	4	15	1	5	5
1	6	5	2	3	7	2	3	3
1	5	4	4	3	15	1	4	4
3	3	3	2	3	4	1	4	4
1	2	3	1	2	16	1	4	3
1	6	5	5	3	9	3	3	3
1	2	3	2	3	9	1	4	4
1	5	5	5	4	12	1	5	4
1	6	5	3	3	10	1	5	5
2	4	3	3	3	9	1	4	3
2	6	5	1	4	5	1	5	5
2	5	2	3	3	17	1	4	3
1	4	2	5	3	15	3	4	4
1	2	3	1	3	7	1	5	5
2	3	4	2	2	5	1	5	5
1	4	2	3	3	5	1	4	4
2	4	4	3	3	9	1	3	4
2	5	5	5	4	12	1	4	4
1	3	3	2	3	9	1	2	2
1	2	5	1	2	3	1	5	5
2	4	3	3	3	10	1	5	5
2	2	3	1	3	15	1	4	4
1	5	3	4	3	16	1	5	5
2	3	3	1	3	16	1	5	4
2	2	4	1	3	9	1	5	4
1	4	2	1	4	12	1	5	5
2	4	3	4	4	16	1	4	4
2	3	3	2	3	3	1	4	4
1	5	5	1	3	9	3	3	3
2	5	3	4	4	1	1	4	4
1	5	5	5	3	5	4	4	4
1	5	3	5	2	12	1	4	4
2	4	4	3	3	17	1	5	5
2	5	3	3	3	1	1	4	4
2	4	2	3	3	7	1	4	4
2	4	3	3	2	7	1	3	3
1	5	5	5	3	7	1	4	4
2	5	3	4	4	18	1	4	4
1	5	2	5	4	9	1	4	3
2	3	3	3	3	10	1	4	3
2	4	3	2	3	14	1	4	4
2	4	3	5	3	7	1	4	4
2	5	4	5	3	16	1	4	3
2	3	2	3	3	20	1	4	4
1	4	3	3	4	16	1	4	4

1	2	3	1	3	14	1	5	5
2	5	2	2	3	16	1	4	3
1	6	5	5	2	13	4	4	2
2	5	4	2	3	15	3	4	4
2	3	2	2	3	20	1	4	4
1	5	2	3	3	7	1	4	4
2	5	3	5	3	12	1	5	4
2	4	3	1	3	12	1	1	1
2	5	5	5	3	7	1	4	4
1	3	3	3	3	9	1	4	4
2	4	4	3	4	9	1	4	4
2	5	4	2	3	9	1	4	4
2	4	4	5	3	9	1	4	4
2	5	5	5	3	9	1	4	4
2	5	5	5	3	9	1	4	4
2	5	5	5	3	9	1	4	4
2	2	3	1	3	9	1	4	3
2	2	5	2	3	9	3	4	4
1	4	2	2	2	14	1	4	4
2	2	3	2	3	9	1	5	5
1	5	2	4	4	4	1	3	3
1	5	2	2	3	13	1	5	4
2	3	4	3	2	20	1	4	3
1	2	3	1	3	20	1	5	5
2	5	2	1	3	9	1	4	4
1	6	5	1	3	9	1	4	4
2	4	3	4	3	12	1	4	3
2	4	4	3	3	9	1	4	4
2	5	2	1	3	9	3	4	3
1	2	4	1	3	5	1	5	5
2	2	4	1	3	13	1	5	5
2	5	3	5	3	13	1	3	3
2	4	2	5	3	7	1	2	2
1	6	2	5	2	8	3	4	4
1	4	3	5	3	9	1	3	3
2	4	4	2	3	13	1	3	3
1	5	4	5	4	15	1	5	5
1	6	5	2	3	8	1	4	4
1	1	5	1	3	4	1	4	4
1	2	4	1	4	13	1	5	5
2	5	2	3	3	9	1	4	4
1	3	4	1	3	2	1	4	4
2	5	2	1	3	9	1	3	3
2	4	2	1	4	8	1	4	4
1	4	2	1	3	3	1	4	4
2	4	4	2	4	15	1	4	4
2	4	4	3	3	20	1	4	4
1	6	5	5	2	8	3	2	2
2	5	3	5	3	9	1	3	3
2	5	4	5	3	16	1	4	3

1	5	5	1	3	12	1	5	4
2	5	3	3	3	13	1	5	4
1	5	4	3	3	4	1	4	4
2	3	3	2	2	15	3	4	4
2	4	3	3	4	5	1	4	4
2	4	4	3	3	9	1	4	4
2	4	3	1	3	12	1	1	1
1	5	5	2	3	20	1	5	4

ITU3	PU1	PU2	PU3	PU4	PEOU1	PEOU2	PEOU3	RP1.1
5	4	4	4	4	4	3	4	4
4	4	4	4	4	4	4	4	5
5	3	4	4	4	3	4	4	4
5	3	3	3	4	3	3	3	4
5	5	5	5	5	5	5	5	5
3	3	2	3	3	4	3	3	4
4	4	4	4	4	3	3	3	5
4	4	4	4	4	3	3	4	4
3	3	3	3	3	3	4	3	3
3	3	3	3	3	3	3	3	3
4	4	4	3	3	4	4	4	5
4	3	3	3	4	2	3	3	4
5	5	5	5	5	5	5	5	2
4	3	4	4	3	2	3	3	4
5	5	5	5	5	5	5	5	2
4	4	4	4	4	3	3	3	4
4	4	4	4	4	3	4	4	2
5	5	5	5	5	5	5	5	4
5	5	5	5	5	5	5	5	2
4	3	4	3	3	3	3	4	5
3	3	2	3	3	1	3	3	5
4	4	4	4	4	4	3	4	3
2	2	2	2	2	3	3	3	4
5	5	5	5	5	5	5	5	1
5	4	4	4	4	3	4	3	4
4	4	4	2	4	4	4	4	3
5	4	4	4	4	4	3	3	3
5	5	5	5	5	1	3	4	1
5	5	5	4	5	2	4	4	4
5	3	5	3	5	4	3	4	2
4	4	4	4	4	4	4	4	2
4	4	4	4	4	4	4	4	2
4	4	4	3	3	3	3	3	3
4	4	4	4	4	4	4	4	2
4	4	4	4	4	2	2	2	5
4	4	4	4	3	4	4	4	3
5	4	4	4	5	3	3	5	4
4	4	4	4	4	4	4	4	4
4	4	4	4	4	4	4	4	3
3	2	3	2	4	2	3	3	2
4	4	4	4	4	4	3	2	3
4	4	4	4	4	4	5	3	3
4	3	4	4	4	3	3	3	4
4	3	4	3	3	3	4	3	3
4	4	3	3	4	3	3	4	2
4	4	4	4	4	3	3	3	4
4	4	4	4	4	4	4	4	4
4	4	4	4	4	4	4	4	4
4	2	2	2	3	4	4	5	3

5	5	5	5	5	4	5	5	1
4	3	4	3	3	2	3	3	4
4	2	4	3	4	2	2	2	3
4	4	4	4	4	4	4	4	2
4	4	4	4	4	4	4	4	4
4	4	4	4	4	3	3	4	3
5	5	5	5	5	4	5	4	3
1	1	1	1	1	1	1	1	1
4	4	4	4	4	4	4	4	1
4	3	3	3	3	2	4	4	1
4	4	4	4	4	2	3	3	1
4	4	4	4	4	4	4	4	1
4	3	3	3	3	3	3	3	1
4	4	4	4	4	3	3	4	1
4	4	4	3	4	2	4	4	1
4	4	4	4	5	2	4	4	1
4	4	4	3	3	4	4	4	1
4	4	4	4	4	3	3	3	1
4	4	4	4	4	4	4	4	1
5	5	5	5	5	5	5	5	1
4	3	3	3	3	4	4	4	1
4	5	4	5	4	3	4	5	1
4	4	4	4	3	2	3	2	1
5	4	5	4	4	3	4	5	1
4	4	4	4	4	2	2	2	1
5	4	4	4	4	3	3	3	1
3	3	3	3	3	2	2	2	1
4	4	4	4	4	2	3	3	1
3	4	4	4	4	3	3	3	1
5	5	5	5	4	4	5	3	1
5	5	5	5	5	3	4	2	1
3	3	3	3	3	3	3	3	1
2	2	2	2	2	3	3	4	1
4	4	4	4	4	3	3	3	1
4	3	3	3	3	4	4	3	1
4	4	4	4	4	3	3	4	1
5	5	5	5	5	5	5	5	1
4	4	4	4	4	4	4	4	1
3	4	4	4	5	2	4	4	1
5	4	3	4	4	5	5	5	4
4	4	4	4	4	4	3	4	1
5	4	4	4	4	3	4	4	1
3	3	3	3	3	1	1	1	1
4	5	4	5	4	3	4	3	1
5	4	3	3	4	4	4	5	1
4	4	4	4	4	3	4	4	1
4	3	4	3	4	3	4	4	1
2	1	1	1	1	1	2	2	1
3	3	4	3	3	3	3	3	4
4	3	3	3	3	2	3	3	5

4	3	3	3	3	3	3	4	4
4	5	5	5	5	5	4	5	2
4	4	4	4	4	4	4	4	1
4	4	4	3	3	4	4	4	1
4	4	4	4	4	4	4	4	1
4	2	4	4	4	2	3	3	1
4	1	1	4	4	4	4	4	1
4	4	4	4	4	4	4	4	1

RP1.2	RP1.3	NP1.1	NP1.2	NP1.3	CP1.1	CP1.2	CP1.3	RP2.1
3	3	4	4	4	3	3	3	2
5	5	5	5	5	5	5	5	1
3	3	4	4	3	3	2	2	2
4	3	4	4	2	3	3	3	2
4	4	3	3	4	5	5	5	5
4	3	3	4	1	3	3	3	3
4	4	4	4	4	1	1	1	1
4	4	4	4	4	3	4	3	3
3	3	3	3	3	4	4	4	2
3	3	3	3	3	3	3	3	3
4	4	4	4	4	2	2	2	2
2	3	4	3	4	3	4	3	2
3	3	5	5	5	5	5	3	2
3	3	4	4	4	2	2	2	2
2	2	4	4	4	3	3	3	5
3	3	2	4	4	2	2	3	2
2	1	3	3	4	4	4	3	4
5	4	5	5	5	4	2	2	1
2	2	5	5	5	5	5	5	2
5	5	4	3	3	3	3	3	2
4	4	3	4	3	3	2	2	3
4	5	3	3	3	3	3	2	3
4	4	3	3	3	2	2	2	3
1	1	5	5	5	5	5	5	4
3	3	4	4	4	4	4	5	2
3	3	4	4	4	4	4	4	3
2	2	4	4	2	4	3	4	1
2	2	5	5	5	5	4	3	1
4	4	4	4	4	2	2	2	2
2	1	5	4	1	5	5	5	2
2	2	4	4	4	4	4	4	3
2	2	4	4	4	4	4	4	1
4	3	3	4	4	3	3	4	2
4	4	3	4	4	3	4	4	3
5	5	4	4	3	3	2	3	2
3	3	4	4	4	4	3	3	3
1	2	4	3	2	3	2	1	3
4	4	4	4	4	3	3	3	3
3	2	4	4	4	5	5	5	1
2	2	5	5	4	5	5	5	2
4	3	4	4	4	4	4	4	3
4	4	2	3	4	3	3	3	3
3	3	4	4	4	3	3	3	2
2	3	4	3	3	3	4	3	2
2	2	4	3	3	3	4	4	3
4	3	4	4	4	3	2	2	3
4	4	4	4	4	3	2	2	4
4	4	4	4	4	4	4	4	2
4	2	4	4	2	2	2	2	2

1	1	5	5	5	5	5	5	1
4	4	4	4	3	4	4	4	3
3	3	3	3	3	3	3	3	2
2	2	4	4	4	3	3	3	2
4	4	4	4	4	4	4	4	4
3	3	4	4	4	4	3	3	3
4	4	5	5	5	3	3	3	2
1	1	1	1	1	1	1	1	2
2	2	4	4	4	4	4	4	2
3	3	4	4	4	5	4	4	3
4	2	3	2	2	2	2	2	2
5	5	2	4	4	1	1	1	2
2	2	2	2	2	2	2	2	2
3	2	3	3	4	4	3	3	2
4	4	2	3	3	3	3	3	3
2	2	3	3	3	3	3	3	3
3	3	4	4	2	4	3	3	3
4	4	4	4	4	3	3	3	2
2	2	4	4	4	4	4	4	1
4	4	3	3	3	3	3	3	2
3	3	3	3	3	3	3	3	3
3	4	4	3	4	3	4	3	3
3	2	2	2	2	2	2	3	3
3	3	5	5	5	4	5	5	2
4	3	3	3	4	4	3	3	2
3	4	4	4	4	4	4	3	2
4	4	4	4	3	3	4	3	3
4	4	4	4	4	2	2	2	2
3	3	3	4	3	3	3	3	2
2	3	4	5	4	4	5	4	2
2	2	5	5	5	4	4	4	4
3	3	3	3	3	3	3	3	3
3	3	2	2	3	5	5	5	3
2	2	4	4	3	3	3	3	2
3	3	4	4	1	4	4	4	3
3	2	2	4	2	3	3	2	3
2	2	3	4	4	5	5	5	2
2	2	3	4	2	4	4	4	3
2	2	4	4	3	5	3	3	2
4	4	4	4	4	4	4	4	3
3	5	3	3	4	4	3	2	2
3	3	5	5	4	4	4	4	2
4	4	2	3	2	3	2	2	2
2	3	4	4	4	4	3	3	2
3	3	4	4	4	3	3	3	2
3	3	3	3	2	3	3	3	2
4	4	3	4	4	3	3	3	1
3	3	3	3	3	3	3	3	3
4	2	3	3	3	3	3	3	3
5	5	4	4	2	4	2	2	2

3	3	3	4	4	4	3	3	2
1	2	4	3	3	4	4	3	2
5	5	3	3	4	3	2	2	2
3	3	4	4	4	3	3	3	3
2	3	3	3	1	4	4	3	1
3	3	4	4	4	2	2	2	2
4	4	4	4	4	2	4	4	4
4	4	4	4	4	4	4	4	4

RP2.2	RP2.3	NP2.1	NP2.2	NP2.3	CP2.1	CP2.2	CP2.3	INN1
2	2	4	4	4	4	4	4	4
1	1	4	4	4	4	4	4	5
2	3	4	4	4	4	4	4	4
2	2	4	4	2	5	5	5	3
1	1	1	4	4	5	5	5	5
3	2	4	4	3	4	4	4	3
1	1	5	5	5	5	5	5	5
3	3	4	4	4	4	4	4	4
2	2	3	3	4	4	4	4	3
3	3	3	3	3	3	3	3	4
2	2	4	4	4	4	3	3	4
1	1	4	4	5	4	5	4	5
1	1	5	5	5	5	5	5	5
2	2	4	4	4	4	4	4	4
1	1	5	5	5	5	5	5	5
2	2	4	4	3	2	4	4	4
2	2	4	4	4	4	4	4	5
1	1	4	4	5	5	5	5	4
2	2	5	5	5	5	5	5	4
2	3	3	3	3	4	4	3	4
3	3	3	3	3	3	3	3	4
3	2	4	4	4	4	4	3	4
3	3	3	3	3	4	4	4	4
4	4	4	4	4	4	4	4	5
2	2	4	2	4	5	5	5	4
2	2	4	4	2	4	4	4	3
1	1	4	4	4	4	4	5	3
1	1	5	5	5	4	5	4	2
2	2	4	4	4	4	4	4	5
1	1	5	5	5	5	5	5	5
3	3	3	3	3	3	3	3	4
1	1	4	4	4	4	4	4	4
2	2	4	4	4	4	4	4	3
2	3	4	4	4	4	4	4	4
2	2	4	4	3	4	4	4	2
3	3	4	4	4	4	4	4	4
2	1	4	5	5	5	5	5	5
3	3	3	3	3	4	4	4	4
1	1	5	5	5	5	5	5	5
2	3	4	4	4	4	4	4	3
2	2	4	4	4	4	4	4	4
2	2	4	4	4	4	5	5	5
2	2	4	4	4	5	5	5	3
3	3	4	4	3	4	4	4	4
2	2	4	4	4	4	4	4	4
3	3	4	4	4	4	4	4	4
4	2	4	4	4	4	4	4	4
2	2	4	4	4	4	4	4	4
2	2	4	4	4	4	4	4	4

1	1	5	5	5	5	5	5	5
2	2	4	4	2	4	4	4	4
2	1	4	3	4	4	4	3	3
2	2	4	4	4	4	4	4	4
4	4	4	4	4	4	4	4	4
3	3	4	4	4	3	3	3	4
1	1	5	5	5	5	5	5	3
2	2	2	2	2	2	2	2	4
2	2	4	4	4	4	4	4	4
3	2	4	5	4	5	5	5	5
2	2	4	4	4	4	4	4	3
2	2	4	4	4	4	4	4	4
2	2	4	4	4	4	4	4	4
3	2	4	4	4	4	4	4	4
2	2	4	4	4	4	4	4	4
2	2	4	4	3	2	2	2	4
2	2	4	4	2	3	3	2	3
2	2	4	4	4	4	4	4	3
1	1	5	5	5	5	5	5	5
2	2	3	4	3	4	4	4	5
3	3	3	3	3	3	3	3	5
2	1	5	4	5	5	5	5	5
3	2	3	4	4	3	4	3	4
3	3	5	5	5	5	5	5	5
2	2	4	4	4	4	4	4	3
2	2	4	4	2	4	4	4	4
3	3	3	3	3	3	3	3	4
2	2	4	4	4	4	4	4	4
2	2	4	4	4	4	4	4	4
1	2	4	4	4	4	4	4	4
2	2	5	5	4	4	4	4	4
3	3	3	3	3	3	3	3	3
3	3	3	3	3	2	2	2	4
2	2	4	4	4	4	4	4	4
3	3	3	3	3	2	3	4	4
3	3	4	4	3	4	4	4	4
1	1	4	4	4	5	5	5	5
4	3	5	4	4	4	4	4	4
1	1	4	4	4	5	5	5	4
2	3	4	4	4	5	5	5	5
2	2	3	3	2	4	4	4	5
2	2	4	4	4	4	4	4	4
2	3	4	3	4	4	4	4	4
2	2	4	4	4	5	4	4	4
2	2	4	4	4	5	5	5	4
2	2	4	4	4	4	4	4	4
2	1	4	4	4	4	4	4	4
3	3	3	3	3	3	3	3	4
3	3	3	3	3	3	3	3	3
2	2	4	4	4	4	4	4	4

2	1	4	4	4	4	5	4	5
2	2	4	4	4	4	4	4	4
3	3	4	4	4	4	4	4	4
3	3	4	4	4	4	4	4	4
1	1	4	4	4	4	4	4	4
2	2	4	4	4	4	4	4	4
4	4	4	4	4	4	4	4	4
4	4	4	4	4	4	4	4	4

INN2	INN3	RISK1	RISK2	RISK3	RISK4	RISK5	RISK6	RISK7
4	4	4	3	4	4	3	2	3
5	3	4	4	4	3	1	1	3
5	2	2	4	2	4	4	3	4
3	2	4	4	4	4	2	2	3
5	5	5	5	5	4	4	2	2
3	3	2	3	4	3	3	3	3
5	5	5	5	5	3	2	3	4
4	4	4	4	4	4	4	4	4
4	3	3	4	4	4	3	2	3
3	3	3	3	3	4	4	4	4
4	3	4	4	4	4	3	2	2
4	3	4	4	4	3	3	3	2
5	5	4	4	5	2	3	1	3
4	3	3	4	4	3	3	2	3
4	4	3	4	4	2	3	2	3
4	3	4	4	4	4	2	2	2
5	5	4	4	5	3	3	2	3
4	4	4	4	4	2	2	1	1
5	5	4	4	5	2	2	2	2
4	2	1	1	2	5	4	3	3
4	4	3	4	4	4	4	3	3
4	4	4	4	4	3	3	3	3
4	3	1	1	1	4	4	4	4
5	5	5	5	5	2	2	1	1
4	4	4	4	4	2	2	2	2
4	4	4	2	2	3	3	3	2
4	3	4	4	4	3	3	3	3
5	2	4	4	3	1	1	1	2
5	3	4	4	4	4	3	3	4
5	4	4	4	4	3	3	3	3
4	2	4	4	4	2	3	3	3
4	4	4	4	4	3	3	3	3
4	3	4	4	4	3	4	3	3
4	2	2	2	2	2	3	2	4
5	3	3	2	2	3	3	3	3
4	4	4	4	4	3	2	2	3
5	5	4	4	5	2	3	1	4
4	3	3	3	3	3	3	3	3
5	5	4	4	4	2	2	2	2
3	2	2	2	2	4	3	5	3
4	4	4	4	4	1	1	3	3
5	4	4	4	4	4	3	2	3
4	3	3	4	4	4	4	3	3
4	3	4	4	4	3	3	3	3
4	3	4	4	4	3	3	3	3
4	3	4	4	4	3	3	3	3
4	3	3	3	4	4	4	4	4
4	3	4	4	4	3	3	3	3
3	5	2	2	2	4	4	2	5

5	3	5	5	5	1	2	2	1
4	3	4	4	4	4	4	3	4
3	2	3	2	3	3	4	4	4
4	3	3	3	3	2	2	2	2
4	3	4	4	4	3	3	3	3
4	3	4	4	4	3	3	3	4
4	1	4	5	5	1	3	1	1
4	3	4	4	4	3	3	3	3
4	3	4	4	4	3	3	3	3
4	4	3	3	3	4	1	1	2
4	2	3	3	4	4	2	2	2
4	3	4	4	4	3	2	2	2
4	3	3	4	4	3	3	3	3
4	4	5	4	4	4	3	3	3
4	4	4	4	4	3	3	3	3
4	4	4	4	4	3	3	2	3
4	3	4	4	4	2	3	4	2
4	2	3	3	3	3	3	2	2
5	5	4	4	4	3	3	2	3
5	3	4	4	4	3	3	3	3
5	5	4	4	4	3	3	3	3
5	2	1	2	2	2	4	3	2
4	2	3	4	3	2	2	1	3
5	5	4	4	4	4	3	3	4
4	2	2	2	3	4	2	2	2
4	3	4	4	4	3	3	3	3
3	3	2	3	3	4	4	4	4
4	2	4	4	4	4	4	3	2
4	3	3	3	3	5	3	3	1
5	3	4	4	5	2	2	1	2
5	5	5	5	5	4	4	2	2
3	3	2	2	2	5	5	5	5
4	3	4	4	4	3	3	3	3
4	3	4	4	4	3	3	3	3
4	3	4	4	4	3	3	3	3
4	3	3	3	4	3	3	2	3
5	5	5	5	5	2	2	2	2
4	3	4	4	4	3	3	3	3
4	3	4	4	4	4	2	2	2
5	5	4	5	4	5	2	1	4
5	5	4	4	4	4	4	4	3
4	2	4	4	4	2	2	3	5
4	3	4	4	4	3	3	3	3
4	3	4	4	4	2	2	2	3
4	4	4	4	4	2	2	3	2
4	3	3	3	4	2	3	3	3
4	4	3	4	4	4	3	3	3
4	2	2	2	2	4	4	4	5
3	3	3	3	3	4	4	4	3
4	3	4	4	4	4	3	2	2

5	3	4	4	4	3	4	3	2
4	3	4	4	4	3	3	3	3
5	4	3	3	3	3	3	4	3
4	4	3	3	3	3	3	3	3
4	3	3	3	3	2	3	2	2
4	3	4	4	4	3	3	3	3
4	3	4	4	4	3	3	3	3
4	3	4	4	4	3	3	3	3

RISK8	RISK9	RISK10	COMP1	COMP2	COMP3	COMP4	COMP5	COMP6
4	1	1	5	5	5	5	5	5
4	1	1	5	5	5	5	5	5
5	2	4	5	5	5	3	3	5
4	3	2	5	5	5	5	4	4
4	1	1	5	5	5	5	5	5
3	3	4	3	4	4	3	3	3
4	4	4	4	4	3	3	4	5
5	3	4	4	4	4	4	4	4
3	3	3	4	4	4	4	4	4
4	4	4	4	4	4	4	4	4
4	2	4	5	5	5	4	4	4
2	2	2	4	4	4	4	4	4
3	5	3	5	4	4	4	4	4
4	3	2	4	4	4	4	4	4
3	1	3	5	5	5	4	4	4
4	3	3	5	4	4	4	4	3
2	1	3	5	5	5	4	4	4
3	1	2	5	5	5	5	5	5
4	1	4	5	4	4	4	4	4
4	3	4	5	4	4	4	4	3
4	3	3	5	5	5	3	3	5
4	2	2	4	4	4	3	3	4
4	3	3	4	4	4	4	4	4
2	1	2	5	5	5	5	5	5
2	2	3	5	4	3	4	4	3
2	2	3	4	4	4	4	5	4
3	3	4	4	4	4	3	3	4
1	3	4	5	5	5	4	4	4
4	3	3	5	5	5	3	3	5
3	2	2	5	5	5	5	5	5
4	2	4	4	4	4	4	4	4
3	3	4	4	4	4	4	4	4
4	4	4	4	4	3	3	3	4
4	3	4	5	5	4	3	3	5
3	4	4	3	2	3	3	3	2
3	3	3	4	4	4	4	4	4
5	1	3	5	5	5	4	5	5
3	3	3	4	4	4	3	3	4
4	2	2	5	5	5	5	5	5
3	5	4	4	4	4	3	3	3
3	2	5	4	4	4	4	4	4
4	2	4	5	5	5	5	5	5
3	3	3	5	5	5	4	4	5
3	3	3	4	4	4	4	4	4
3	3	3	4	4	4	4	4	4
3	3	3	4	4	4	4	4	4
4	4	4	4	4	4	4	4	4
4	3	4	4	4	4	4	4	4
5	2	4	5	5	5	4	4	5

2	1	2	5	5	5	5	5	5
4	3	3	5	5	5	5	5	5
3	5	4	3	3	2	1	3	4
2	3	3	4	4	4	4	4	2
3	3	3	4	4	4	4	4	4
4	3	3	4	4	4	4	4	4
3	2	1	5	5	5	5	5	4
3	3	3	4	4	4	4	4	4
3	3	3	4	4	4	4	4	4
4	3	3	1	2	1	2	2	4
2	3	2	4	3	3	2	2	2
4	2	4	4	5	5	3	3	5
3	3	3	4	4	4	4	4	4
3	4	4	4	4	4	4	4	4
3	4	3	4	4	4	4	4	4
3	2	3	4	2	4	4	4	4
3	2	4	5	5	5	5	5	3
2	2	2	2	4	4	4	4	4
4	2	2	5	5	5	5	5	5
3	3	3	5	5	5	5	5	5
3	3	3	4	4	4	4	4	4
2	2	2	5	5	5	3	5	5
2	2	1	3	4	4	4	4	3
4	2	3	5	5	5	5	5	5
3	4	3	2	2	3	3	3	3
3	4	3	4	4	4	3	3	3
4	4	3	2	2	2	2	2	3
4	3	4	4	4	4	4	4	4
4	3	3	5	5	5	3	2	3
4	3	3	5	4	4	3	4	5
4	1	2	5	5	5	5	5	5
5	5	5	3	3	3	3	3	3
3	3	3	4	4	4	4	4	4
3	3	3	4	4	4	4	4	4
3	3	3	4	4	4	4	4	4
3	3	3	4	3	4	3	3	3
3	1	1	5	5	5	5	5	5
3	3	3	4	4	4	4	4	4
4	1	3	5	5	5	2	2	4
5	1	2	5	5	5	4	4	5
3	2	2	4	4	3	4	4	4
5	3	4	5	4	4	4	4	4
3	3	3	4	4	4	4	4	4
3	2	3	4	4	3	3	2	4
3	2	2	5	5	5	4	5	5
3	3	3	4	4	2	4	4	4
4	3	3	5	5	5	4	4	4
5	3	3	5	5	3	2	2	4
3	3	4	4	4	4	3	3	3
4	3	3	4	2	4	4	4	4

3	2	2	5	5	5	4	3	3
3	3	3	4	4	4	4	4	4
4	1	3	5	5	5	5	5	5
3	3	4	3	3	3	3	3	3
3	2	2	5	4	5	5	5	5
3	3	3	4	4	4	4	4	4
3	3	3	4	4	4	4	4	4
3	3	3	4	4	4	4	4	4

COMP7	COMP8	COMP9	FUN1	FUN2	PZ1	PZ2
5	5	5	5	5	5	5
5	5	5	5	5	4	4
5	4	5	4	4	4	4
5	5	4	5	3	5	4
5	5	5	5	5	5	5
3	3	3	4	3	3	3
4	3	4	5	4	4	4
4	4	4	4	4	4	4
3	3	4	4	4	4	4
4	4	4	3	3	4	4
4	4	3	4	4	4	4
4	4	3	5	4	4	4
4	4	4	4	4	4	4
4	4	3	4	4	4	4
4	4	4	4	4	5	5
4	4	4	2	4	4	4
4	4	4	4	4	3	3
5	5	5	5	5	5	5
4	4	4	4	5	4	4
4	4	4	4	4	4	4
4	4	3	4	3	4	5
4	4	4	4	4	4	3
4	4	4	4	4	4	4
5	5	5	5	5	3	3
4	4	4	5	5	5	5
4	4	4	4	4	4	4
4	4	4	4	4	4	4
2	5	4	5	3	5	5
5	5	5	5	5	5	5
5	5	5	5	5	5	4
4	4	4	5	5	5	5
4	4	4	4	4	2	4
3	4	3	4	4	4	4
4	4	3	4	4	4	5
2	2	2	4	4	3	3
4	3	4	4	4	4	4
5	5	5	5	5	5	5
4	4	4	4	4	4	4
5	5	5	5	5	5	5
3	3	3	3	3	4	4
4	4	4	4	4	5	5
5	5	5	4	4	5	5
4	4	3	5	5	5	5
4	4	4	4	4	4	4
4	4	4	4	4	4	4
4	4	4	4	4	4	4
4	4	4	4	4	4	4
4	4	4	4	4	4	4
5	5	5	4	2	4	5

5	5	5	5	5	5	5
4	4	5	4	3	3	4
3	2	3	4	4	2	4
3	4	3	4	4	4	4
4	4	4	4	4	4	4
4	4	4	4	4	4	4
4	5	5	5	4	5	5
4	4	4	4	4	4	4
4	4	4	4	4	4	4
4	4	4	5	4	4	4
4	3	2	4	4	4	4
4	5	2	5	5	5	5
4	4	3	4	4	4	4
4	4	4	4	5	5	5
4	4	4	4	4	4	4
3	4	5	4	4	4	4
4	4	3	5	4	4	4
4	4	4	4	4	4	4
5	5	5	4	4	4	4
5	5	5	5	5	5	4
4	4	4	5	5	5	5
4	4	4	5	5	5	5
3	4	5	5	4	3	3
5	5	5	5	5	5	5
3	3	3	4	4	4	4
3	3	3	4	4	4	4
3	3	3	3	3	3	3
4	4	4	4	3	4	4
4	4	3	5	5	3	3
4	4	4	5	5	5	5
5	5	5	5	4	3	2
3	3	3	3	3	3	3
4	4	4	4	4	4	4
4	4	4	4	4	4	4
4	4	4	4	4	4	4
3	4	4	4	4	4	4
5	5	5	5	5	5	5
4	4	4	4	4	4	4
5	5	3	4	4	4	4
5	5	5	5	5	5	5
4	4	4	4	4	4	4
4	4	4	5	5	4	5
4	4	4	4	4	4	4
4	3	3	4	4	4	4
4	5	4	4	4	4	4
4	4	4	4	4	4	4
4	4	4	5	4	4	4
3	4	4	3	3	2	2
3	3	3	3	3	3	3
4	4	5	4	4	4	4

3	3	3	5	5	4	4
4	4	4	4	4	4	4
3	4	4	4	4	4	4
3	3	3	4	4	4	4
5	5	5	5	5	5	5
4	4	4	4	4	4	4
4	4	4	4	4	4	4
4	4	4	4	4	4	4
